# Public Acceptance of Plant Biotechnology and GM Crops

**DOI:** 10.3390/v7082819

**Published:** 2015-07-30

**Authors:** Jan M. Lucht

**Affiliations:** Scienceindustries, Swiss Business Association Chemistry Pharma Biotech, P.O. Box 1826, Zurich CH-8021, Switzerland; E-Mail: lucht@mac.com; Tel.: +41-44-368-17-63

**Keywords:** farmers, consumers, risk/benefit perception, knowledge, trust, personal attitudes, politics, GMO labeling, new breeding techniques

## Abstract

A wide gap exists between the rapid acceptance of genetically modified (GM) crops for cultivation by farmers in many countries and in the global markets for food and feed, and the often-limited acceptance by consumers. This review contrasts the advances of practical applications of agricultural biotechnology with the divergent paths—also affecting the development of virus resistant transgenic crops—of political and regulatory frameworks for GM crops and food in different parts of the world. These have also shaped the different opinions of consumers. Important factors influencing consumer’s attitudes are the perception of risks and benefits, knowledge and trust, and personal values. Recent political and societal developments show a hardening of the negative environment for agricultural biotechnology in Europe, a growing discussion—including calls for labeling of GM food—in the USA, and a careful development in China towards a possible authorization of GM rice that takes the societal discussions into account. New breeding techniques address some consumers’ concerns with transgenic crops, but it is not clear yet how consumers’ attitudes towards them will develop. Discussions about agriculture would be more productive, if they would focus less on technologies, but on common aims and underlying values.

## 1. Growth of Agricultural Biotechnology and Technology Adoption by Farmers

About a decade after the first transgenic plants were created in the laboratory, genetically modified food plants were introduced into the market. In 1994, the FLAVR SAVR tomato, modified to delay premature fruit softening, hit the shelves of U.S. grocery stores [1]. From 1996 on, insect resistant Bt maize and Bt cotton, as well as transgenic herbicide tolerant soybeans and oilseed rape were planted on rapidly increasing areas. In 2014, genetically modified (GM) crops were grown by 18 million farmers in 28 countries on a total surface of 181.5 million hectares, which correspond to already 13% of the world’s arable surface. This rapid increase makes biotech crops the fastest adopted crop technology of the last decades. Globally, 82% of the total crop area for soybeans, 68% for cotton, 30% for maize and 25% for oilseed rape were planted with GM varieties in 2014 [2].

In many countries where farmers are free to choose which technologies they want to employ, GM plants have superseded conventional varieties. For maize, cotton and soybean in the U.S., the adoption rate for biotech varieties is well above 90%. The same holds true for soybeans in Brazil and Argentina, cotton in India and China, and oilseed rape in Canada [2]. Especially impressive was the speed of adoption for transgenic herbicide tolerant sugar beets in the U.S., which—because of limited availability of seeds—were grown in small areas, only in 2006 and 2007. Then, when sufficient seed became available, adoption of the GM varieties skyrocketed in just two years to 95% in 2009, and has increased even further since then. Farmers benefit from facilitated weed control with herbicide tolerant sugar beet, a reduced number of herbicide treatments, which saves both time and expense, and higher profits, which explains why so many farmers switched to the new biotech varieties in such a short time [3]. Transgenic, papaya ringspot-virus (PRSV) resistant papaya trees were introduced in Hawaii in 1998 after the papaya production was on the verge of collapse because of a devastating outbreak of PRSV infections. They were rapidly taken up by the large majority of the papaya farmers in almost 90% on the papaya cultivation surface in Hawaii, and are credited with saving the Hawaii papaya industry from extinction [4].

### 1.1. Reasons for Adoption and Acceptance of GM Crops for Farmers

Reasons for farmers to select biotech over conventional crop varieties have been extensively studied. A recent meta-analysis of 147 agronomical studies by Klümper and Quaim in 2014 [5] looked at the performance of a variety of GM corps in different world regions, in different agricultural systems, in developing and in industrialized countries. Farmer’s profits increased by 68% on average when using biotech crops. Crop yields rose by 22%, the expense for pesticides declined by 39%. The reported increases for yield and profit were generally higher for developing countries than in developed countries. Thus, despite of higher seed cost for biotech varieties, farmers profit financially by planting them. In addition to these economic advantages, farmers often cite non-monetary benefits, such as time savings, ease of use, and more flexibility in their planning [6,7,8,9]. A positive side-effect of the switch to biotech plant varieties has been a pronounced reduction in insecticide quantities used on insect-resistant Bt-crops (−41.7%), and the possibility to switch to more environmentally benign herbicides with herbicide tolerant crops [5].

### 1.2. Important Role for GM Crops as Feed in Many Countries

About 70% to 90% of the globally produced GM cops are used as feed for food-producing animals. In the USA itself, with a high adoption of GM crops, more than 95% of food-producing animals consume GM feed. During the last decade alone, this corresponded to more than 100 billion animals. Health and performance of these animals is closely monitored. No detrimental effects of the GM feed *versus* conventional feed were observed when analyzing this very large dataset [10]. The global demand for certified non-GM feed is quite limited. For soybean, the share of this niche market has been estimated to be less than 4.5%, for maize at around 7% of traded commodities. Given the wide adoption of GM varieties in main export countries, more than 90% of the globally traded soybean may contain GM. For the European Union (EU), less than 15% of the about 30 million tons of soybeans and soy products for feed imported each year (more than 60 kg per EU citizen) are identity-preserved certified GM free. The large majority of soy-based animal feed in the EU, thus, contains genetically modified components [10].

### 1.3. GM Crops in Europe

Due to a highly restrictive regulatory environment, just a single GM plant, the insect resistant Bt maize MON810, is authorized for cultivation in the EU. Spain is the only European country with significant plantings of this GM crop. Since its introduction in 1998, famers had good experiences with the efficacy of Bt maize against the corn borer in infested regions, and with its economic performance [11,12]. Meanwhile, more than 30% of the total Spanish maize area is planted with Bt maize. Four other EU countries (Portugal, Czech Republic, Romania, and Slovakia) grow a limited amount of Bt maize. The total GM crop plantings in Europe of 143,000 ha in 2014, however, account for only a small fraction of the global GM crop area [2]. In other European countries, either Bt maize is less relevant to farmer local needs, its cultivation is discouraged by onerous regulations, or is outright banned.

An interesting case was the cultivation of herbicide tolerant GM soybeans in Romania starting in 1999, which gave farmers new weed control options, increased average yields by over 30%, and made this crop the most profitable arable crop grown in Romania. In 2006, the GM soy varieties were grown on 137,000 ha and had reached an adoption rate of 68% compared with the total soy production area. Because of increased yields and plantings, Romania’s soy production soared, and surplus soybeans could be exported to other European countries while soybean meal imports decreased substantially. When Romania joined the European Union in 2007, where GM soybeans do not have a cultivation authorization, farmers were forced to return to conventional seed varieties. Because of strongly reduced profitability, the area planted with soybeans has shrunk by 70% within just two years, Romania became dependent on expensive soybean imports like the rest of Europe, and farmers lost a very profitable crop [13].

Even in European countries without current GM crop cultivation, attitudes of farmers towards the potential of crops improved by biotechnology tend to be quite positive. In a survey, over a half of German farmers and almost a half of Czech and UK farmers indicated that they would be keen on adopting GM herbicide tolerant oilseed rape, which would facilitate weed management. Over a third of Spanish, French, and Hungarian farmers would be interested in adopting GM herbicide tolerant maize [14]. In another study, one half or more of surveyed UK farmers indicated that they would consider growing GM maize, oilseed rape, or sugar beet, if they were licensed by the government [15].

### 1.4. Good Global Acceptance of GM Crops among Farmers and Their Customers

Taken together, the adoption of GM crops by millions of farmers during the last two decades has been rapid and wide-spread in countries where biotech varieties are available, where they address specific farmer needs, and where they can be cultivated without disproportionate restrictions. A significant proportion of globally traded commodities is produced from transgenic plants. Since 1994, 65 countries have issued import and/or cultivation authorizations [2]. For the main global use for GM crops, animal feed, there apparently is good acceptance and a large market acceptance, with only a small sector of the market requiring certified GM-free feed. Even reportedly GM skeptical Europe imports and uses very large amounts of genetically modified feedstuff, which eventually contributes to food production.

## 2. Consumer Attitudes towards GM Crops and GM Food

### 2.1. Divergent Developments between North America and Europe

Despite the positive experience of farmers with biotech crops in many countries, the global trade with commodities derived from them, and the huge import volumes even into countries that do not grow GM crops themselves, marked differences exist between consumer’s opinions about GM crops and food in different world regions. When the first GM crops began to be grown on a large scale in the USA starting in 1996, farmers embraced them and products rapidly penetrated the markets for feed and food. The U.S. authorities had adopted a quite permissive approval policy for GM food products, and have not required GM labeling [16]. Additionally, in the years following their introduction, the majority of U.S. consumers expressed little to no concern about food and agricultural biotechnology, and were likely to buy food products produced from GM plants, although consumer awareness and knowledge about GM food was superficial [17].

The arrival of the first shipments of GM soy in Europe from the U.S. in 1996, on the other hand, was met by intense protests from environmental nongovernmental organizations (NGOs). The development of the regulatory and the societal framework on both sides of the Atlantic has diverged strongly, which had a direct effect also on consumer attitudes [18]. Contrary to the situation in the U.S., the EU adopted increasingly stringent approval [19] and labeling regulations for GM food and feed since 1990. From 1997 on, food prepared from GMO had to be labeled in the EU, if the genetic modification could be detected in the end product. The requirements have become stricter with a focus on process-based labeling [16]. Since 2003, also purified food and feed products derived from GMO, such as oils or sugars, have to be labeled, although they are physically and chemically identical to products derived from non-GM crops [20,21]. Today, the EU has a very stringent regulatory framework for GM crop cultivation or their import for food and feed use [19,22]. Authorizations for GM crop cultivation or imports have become a highly charged political topic, with internal disagreement between EU member states and difficulties in reaching a common European position [23]. This results in long delays in the increasingly politicized decision making, and hampers the EU authorization process for biotech crops. On average, it takes at least 15 to 20 months longer than that in the U.S., Brazil, and Canada, the major feed exporters to the EU, with an increasing backlog. Such asynchronous approvals cause difficulties with the supply, and can disrupt imports, when traces of not-yet authorized GMOs are found in commodities [2].

While NGOs traditionally play a less powerful role in the U.S., they have been very successful in Europe in framing GMOs as threat to biodiversity, farmer autonomy, and food safety [24,25]. Together with supporters from Green political parties and the organic movement, these groups have focused strongly on potential risks and possible negative effects of GM food and feed. Their often-sensationalistic campaigns were taken up and multiplied by media articles [26]. In the population, worries about new technologies intermingled with general concerns about the functioning of society, and a distrust of companies and institutions involved in development and regulation of GM crops, food and feed [16,18,27,28].

### 2.2. European Consumers Attitudes towards GM Crops and Food

Given this societal and political climate, it is not surprising that European consumers generally appear to have more negative perceptions and lower intentions to purchase GM foods in comparison to consumers from North America [29,30,31,32]. In Europe, citizens’ views of a variety of topics are regularly surveyed by the Eurobarometer studies carried out by the European Commission to monitor the evolution of public opinion in the Member States over several years, using uniform questionnaires and large numbers of interviews to obtain representative data [33,34,35]. In general, Europeans seem to have rather positive attitudes in respect to technological developments. When asked for the 2010 Eurobarometer about whether a variety of defined technologies might have a positive effect on the way of life in twenty years, optimistic responses clearly outnumbered pessimistic ones. “Biotechnology and genetic engineering” were expected by 53% of respondents to have a positive effect, while 11% expected no effect, and only 20% expected a negative effect.

This positive general attitude is in contrast to when consumers where asked about the specific application of biotechnology in GM foods. Most respondents were aware of this topic. Only 18% had not heard about it, while 55% had talked about the topic, or actively searched for information. In 2010, 23% of respondents from the EU 27 states thought that GM food should be supported, while 61% disagreed with this view. There seems to be a slight downward trend: five years earlier, in 2005, support still was somewhat higher at 27%, with 57% disagreeing. In fact, GM food has been described as the “black sheep” of biotech [33,35]. A comparison of responses from different EU countries shows clear differences. Spain and Portugal, where Bt maize is grown, are among the countries with the highest GM food approval rate, while countries with GM cultivation bans (like Austria, Germany or France) had only a low approval [33]. Most European consumers thus appear to be quite skeptical concerning GM food, when asked about this topic in isolation. However, in comparison to other perceived food risks, GM foods do not score very highly. When potential benefits of GM food of GM crops are pointed out, support seems to increase [34].

### 2.3. Consumers in Europe: What They Say Is Not What They Will Do

Consumer surveys often are carried out by interviews or questionnaires, or by experimental approaches that do not closely match the real shopping situation, and where consumers are aware that their behavior is being monitored [30]. Especially in Europe, where almost no GM food products are found on the market and consumers do not come into regular contact with them in their daily life, these results do not necessarily predict how consumers would actually behave if they had the choice to buy GM food [36]. A few studies have therefore looked at the behavior of shoppers in real market situations. Given the choice of fruits labeled either “organic”, “conventional” or “spray-free GM” at real roadside fruit stalls in Sweden, France, Belgium, the UK and Germany, consumers preferred the “organic” fruits (in fact, all fruits were identical), but the “spray-free GM” fruits achieved a market share of around 20% when prices for the three varieties were identical. This share soared in most countries when “spray-free GM” fruits were sold at a 15% discount, and the “organic” fruits were sold at a 15% premium to reflect more realistic market prices, rising to 43% in Sweden, 33% in France, 30% in the UK and 36% in Germany [37].

When the revealed preference obtained from the real marketing experiments was compared to the stated preference, obtained by theoretical choices via questionnaires but with the same fruits and prices as in the practical experiments, there was a strong discrepancy. In Germany, only 12% of respondents indicated that they would buy the “spray-free GM” fruit even at the discount; in Sweden the stated preference under these conditions shrunk to 31%. The authors interpret this difference as a result the social stigma that buying GM food has in Europe: “What consumers say they will choose in a survey and what they actually choose in a real-purchase situation may differ substantially when their decision is framed by a socially charged issue such as genetic modification” [38].

Additionally, in Switzerland, practical marketing experiments at market stalls showed that consumers buy GM food when it is offered. Here, shoppers had the choice between maize bread made with organic, conventional or genetically modified Bt maize. The total market share for GM maize bread was 20%; 23% of shoppers bought at least one loaf of GM bread. Interestingly, average sales were about 30% higher when all three kind of maize bread, including the GM maize variety, were on offer, then when only organic and conventional bread were sold. Under the setting of this practical sales experiment, the offering of GM bread side-by side with the other varieties did not deter customers and harm total sales. On the contrary, shoppers apparently like the freedom to choose from a larger variety of different breads [39,40]. Taken together, the practical sale of GM food in Europe shows that—at least under the experimental conditions—a not insignificant minority of consumers there bought it. Additionally, despite a voiced negative attitude toward GM food by a majority of European consumers, over 50% of respondents did not actively avoid purchase of GM foods, even in EU countries where a small number of such products is on the market [36]. The almost complete absence of GM food products in the European market, thus, cannot be explained by unanimous consumer rejection, but rather by marketing decisions of the food retailers [41].

One caveat when interpreting surveys of consumers’ attitudes is the large influence the design of the study and the questionnaire can have on the outcome. Small differences in the wording of questions can induce potentially important differences in the type of answers. In addition, direct comparisons of surveys of consumers’ attitudes from different geographic regions are difficult. Surveys in the EU have more often focused on risks and ethical implications of GM food and crops than those from the U.S., guiding the respondents to think more about potentially problematic aspects of technology, which may have played a role in the apparently more skeptical attitude in Europe [42].

### 2.4. Consumer Attitudes in Other Regions

Compared to the large number of consumer’s GM crop and food attitude studies from Europe and North America, more limited data are available from other geographical regions [32]. Interesting is the situation in China, where the government has invested huge sums in the development of GM crops to aid in meeting the food demands of its large population. GM insect resistant cotton is widely grown in China and has an adoption rate of over 90%. In contrast, despite of advanced research and development (R & D) and also successful field testing of insect resistant Bt rice, the government has been reluctant to grant authorization for commercial planting of this GM food crop [2]. Studies show, that the attitude of consumers in China for GM foods generally was quite positive, especially if they have product-enhancing attributes. Over the last years, however, the number of skeptical consumers appears to have grown, as discussions about GM food have increased [43,44,45,46].

### 2.5. Factors Affecting Consumer Attitudes towards GM Food

Food is a central element of daily life, and hence an important topic for many consumers. New food technologies therefore face close scrutiny, and are often met with skepticism—sometimes described as “food neophobia”. Genetic modification of plants and their use as food or feed is an especially contentious issue. Factors contributing to consumer’s perception and the development of their attitude towards new technologies and GM food, therefore, have been studied by many researchers, using different approaches [47,48,49,50,51].

#### 2.5.1. Perceptions of Risk and Benefits

Risk and benefits perceptions play an important role for consumers behavior [32,48,49,50]. Despite the obvious advantages of GM crops for farmers, which have driven their widespread adoption over the last two decades [5,6,7,9], these benefits are not perceived by consumers [52]. Indeed, the vast majority of GM crops cultivated today have agricultural “input traits” like insect resistance or herbicide tolerance, that facilitate their cultivation for the farmer, but have no perceptible influence on food characteristics or quality. The large-scale cultivation of GM crops brings significant global welfare gains [6]. At the farm level alone, the net economic benefits have been estimated to be $18.8 billion in 2012 [9], but the total economic benefit is spread among farmers, seed and technology providers, and consumers, with the latter reaping a significant fraction (in many cases more than half) of the total benefit [7]. It has been estimated that world market prices for corn, soybeans, and canola would probably be respectively 5.8%, 9.6%, and 3.8% higher, if GM crops would not be available [53], other authors assume even higher effects of the technology on market prices [54]. The lower commodity prices due to the use of GM crops contribute to lower food prices, from which consumers benefit without being aware of it and the connection between market prices and GM crops. Additionally, the significant benefits of GM crops for the environment, due to increased productivity alleviating pressure to expand agricultural surfaces, a reduced environmental footprint of production methods, and a large reduction in greenhouse gas emissions [55,56] are not perceived by most consumers as being advantageous to them [52].

On the other hand, consumers might worry about possible negative effects of GM food on their health, the effects of consumption of “foreign” DNA, unexpected changes in nutritional quality or allergenicity. Although these perceived risks are scientifically unfounded [52], and the research conducted so far has not detected any significant health hazards directly connected with the use of GM crops [57,58], without perceptible benefits consumer’s decisions might still be tipped towards avoidance of GM food. An analysis of data from the Eurobarometer survey series suggest that it is this absence of perceptible benefits, rather than perceived possible risks, that are at the root for low acceptance of GM food [59]. GM products with direct, tangible benefits for the consumer, such as increased content of vitamins or omega-3 fatty acids, blood cholesterol-lowering properties or a reduced requirement for insecticide treatment with fresh vegetables, increase acceptance [8,30,32,46,48]. In many countries, consumers would even be willing to pay a price premium for biofortified GM food [60]. Interestingly, there seems to be a regional difference in the relative perception of risks and benefits of GM foods. While European consumers have a more pronounced risk perception than those in North America and Asia, the reverse is true for benefit perceptions [32]. It remains to be seen whether the introduction of second-generation GM crops with consumer benefits will have a large impact on overall consumer acceptance. A GM soybean variety that produces oil with a healthier fatty acid composition, a GM non-browning apple, and GM potatoes with lower acrylamide content after frying have recently received approvals in some countries, and are beginning to enter the market.

#### 2.5.2. Knowledge and Trust

To make decisions, consumers need information, and many consumers felt or were uninformed about the topic of GM food when it became available [17,48,61,62]. When the first products were introduced into the market and met with skepticism, many experts assumed that this was due to a lack of information about the process of genetic modification itself, its consequences and its merits (“deficit model”). This lack of information was thought to induce uncertainty about risks and benefits of GM foods, and therefore rejection of the technology. Educated with objective information, consumers might weigh risks and benefits in a rational way, and thereby come to more positive attitudes and buying decisions [63]. Indeed, experimental auctions in 2003 with (hypothetical) GM products in three U.S. states, Great Britain and France showed that in many cases, consumer attitudes towards GM food improved after they had received information about positive environmental, health or world food supply effects. The prior attitudes and subjective information of the subjects influenced the reception of the additional information. Where participants held a positive view of GM foods or felt a lack of prior subjective information, the new information was taken up readily and affected the buying behavior. Negative attitudes towards genetic modification of food decreased the willingness to accept new (positive) information. Interestingly, for the test subjects from France, no change in attitude could be observed after the information intervention [62]—possibly the participants already held firm views before the experiment. Similar experiments from the U.S. also showed a strong effect of prior beliefs and information of the possibility to influence consumers’ views by either positive or negative information about GM food. Uninformed subjects were susceptible to information provided from external sources, while informed participants were generally not affected significantly by additional information [64]. This seems also be true for the influence of media on consumers’ attitudes: depending on the level of prior knowledge, media reporting about risks of GM foods can produce major swings in attitudes, or not [26,28].

It has become increasingly clear that information strategies focusing just on the factual aspects do not convince many consumers of the benefits of GM foods. In a large-scale experiment with European consumers, information material about food biotechnology and product examples with health and sustainability benefits failed to change the general attitude of consumers, and product choice was even affected in a negative way [63]. This was explained by the power of pre-existing positions. Apparently, product-oriented attitudes are not formed in a “bottom-up”-process from the mere physical or functional characteristics of the attitude object, but rather in a “top-down”-process, where the beliefs develop embedded in a system of general socio-political attitudes and values. This approach is especially important in situations where consumers feel that they do not have sufficient factual or experiential knowledge. It is unlikely that a pre-existing attitude structure can be changed easily by just providing information [65]. The simplistic “deficit model” is no efficient basis for a strategy to engage in a dialogue with society and possibly change its attitudes [66], rather a more integrative and holistic approach is needed, including the recognition of the importance of societal processes, increased public participation in decision-making, and increasing trust between actors. Basic scientific knowledge, or the results from risk assessments, seem to play a less important part for the acceptance of GM corps and food than metaphors and narratives, since many consumer decisions are strongly influenced by affect, which facilitates complex decision-making processes [49,67].

There seems to be little correlation between formal education and attitudes towards GM food in Europe. In the Eurobarometer surveys, there is no significant difference in the support or rejection of GM food between respondents with or without a family science background or a science degree of their own [33,35,59]. A survey from Switzerland showed that basic knowledge about biology, or more specific knowledge about genetic engineering, did have only a very moderate influence on people’s acceptance of nonmedical uses of genetic engineering [49]. On the other hand, results also from Europe showed that self-reported scientific literacy seems to be linked to the attitudes towards GM foods. People who feel that they are well informed about science, and that they understand GM food, are more likely to support genetically modified foods [68].

Interestingly, a recent survey by the Pew Research Center [69] found striking differences in the U.S. between the public and American Association for the Advancement of Science (AAAS) scientists’ views on whether genetically modified foods are safe to eat. While 88% of AAAS scientists think that eating GM food is safe, only 37% of the general public shares this belief. This 51-percentage point difference between scientists’ and the public’s views was the largest opinion gap of all 13 topics surveyed. It is not clear, however, whether this large difference was due to differences in knowledge, or in the level of familiarity with the way scientists think and work. Apparently, the trust of the general public in scientists in this topic is rather limited: a full 67% of the respondents is convinced that scientists do not have a clear understanding of the health effects of genetically modified crops.

Consumers have only a limited knowledge about new technologies such as genetic engineering, and therefore often feel unable to decide for themselves whether GM crops might carry risks, and how to weigh that against possible benefits. They therefore depend on people they consider trustworthy experts to make informed decisions, which they can use as guide to establish their own positions. Therefore, trust plays an important role in the assessment of GM food by lay persons [47,48,59,70].

Results from the Eurobarometer surveys show that European citizens have high trust in medical doctors, university scientists and consumer organizations, medium trust in media and environmental groups, and comparatively low trust in national governments and the biotech industry. There was a sizeable rise in trust for national governments, environmental groups and the biotech industry between 2005 and 2010, the most pronounced for the industry [33,35]. A very similar picture is seen in the U.S.: here, evaluators (scientists, universities, and medical professionals) are the most trusted, watchdogs (consumer advocacy and environmental organizations, and media sources) receive moderate trust, while merchants (grocers, industry, and farmers) are least trusted [71]. Problematic is the fact, that those organizations with the largest resources and responsibilities to ensure the safety of GM food receive little trust, which might be an obstacle for the acceptance of GM food in general.

#### 2.5.3. Personal Attitudes, Values and Psychological Factors

The perception of new food technologies is strongly affected by a complex and deeply rooted set of personal values and attitudes. A very important factor seems to be the personal importance of the naturalness of a product. New food technologies are evaluated more negatively, when consumers have a strong preference for organic food and a “natural” food production [47]. Attitudes to nature, to technology, to skepticism about new kinds of food, the degree of feelings of alienation from the marketplace and the appraisal of one’s own knowledge strongly influence the perception and processing of factual information, resulting in different outcomes depending on the preexisting value set [65]. Additional factors affecting personal attitudes about GM crops and food are cultural values like an aversion to be invaded or dominated by foreign food culture (“slow food” as opposed to “fast food”), and conflicts with the religious or moral belief system, or the perceived natural order of things. Additionally, personal world views affect the way risks are perceived [51,70]. Moral concerns by consumers, as a factor influencing the attitudes towards GM food, appear to be higher in North America and Asia than in Europe [32]. Many consumers also have socio-economic concerns. They worry about patenting, negative effects of GM crops on small farmers and a possible contribution to global inequality, a growing influence of multinational corporations on the food supply, or feel that large companies are the main beneficiary of agricultural biotechnology, while a possible risk is assumed by the consumer. These negative sentiments are often strongly correlated with an increased perception of health or environmental risks of GM crops and food [22,29,72].

For many people, GMOs and especially their application in agriculture and food, have become a place to focus broader moral concerns about everything they consider bad in modern agriculture and food production. The opposition against modern socio-economic developments, like the increasing globalization, and concerns about the functioning of society in general appear to have crystallized around just one topic, GM crops and food [27,73,74]. This makes discussions about agricultural biotechnology often very difficult and inefficient, since the public debate focuses on scientific pro- and contra-arguments like economic benefits *versus* possible environmental risks, while underlying differences in attitudes and values that are at the heart of the different standpoints are rarely addressed.

## 3. Recent Political and Societal Developments

How is the public acceptance of GM crops and GM food connected with the development of the political and regulatory framework for applications of plant biotechnology, and *vice versa*? Here, one can observe marked differences depending on the geographical location.

### 3.1. Europe

During the last three decades, several food scares and crises (BSE, dioxin, foot and mouth disease, bird flu) have negatively affected public confidence in food production and the safety of food. Food safety, including that of GM food, therefore has become a top priority for European legislative authorities [22]. In response to the perceived consumer’s skepticism and efficient lobbying by environmental NGOs, a very strict authorization system for GM crops and feed/food was introduced in Europe over a decade ago, shaped by regard to the “precautionary principle”. It was accompanied by process-based labeling requirements for all food/feed derived from GM plants, even if no trace of the genetic modification can be detected in the purified end product [19,21,25,72]. GM labeling was supposed to give consumers the possibility to retain their freedom of choice, and allow them to decide whether they wanted to buy products based on GM plants, or avoid them. Although over 60 GM crop events are authorized for import into the EU as food or feed, almost no labeled GM food products are to be found on grocery shelves in most EU countries. This limited market presence does not reflect total refusal of consumers to buy such products—practical sales experiments have shown that a sizable minority European consumers buy correctly labeled GM food, if it is offered [36,37,40]. Rather, retailers want to avoid negative publicity from environmental NGO protests against GM food products, who send “gene detectives” into supermarkets to look for GM labels, and then put strong pressure on shops to immediately remove these legal, authorized and correctly labeled products. Direct product experience has been identified as an important factor for consumer acceptance [65], so the lack of GM food products on the market preserves a preexisting negative attitude. There is little active demand from consumers for GM food products, but even for skeptical consumers the question of GM food plays only a marginal role, since such products do not play a role in their everyday life [36]. In contrast to these “weak attitudes” of most consumers, NGO pressure groups display intense resistance against GM foods on the market. Although they are a vociferous minority, retailers behave in an opportunistic way to avoid any problems, and follow the “strong attitudes” [41].

In this general climate of distrust against biotech crops, also research suffers. Regular destructions of field trials of GM plants by vandals have made field research with GM plants virtually impossible in many European countries, the once strong number of field trials is dwindling [75,76], and scientists’ willingness to publicly support plant genetic engineering is decreasing. Due to the unfavorable conditions, major plant biotechnology companies have moved research and development activities away from Europe and closer to the booming markets on other continents, and even have withdrawn requests for cultivation authorizations for GM crops in Europe [77,78,79]. A dramatic example was the decision by the large multinational company BASF, in 2012, to close its European plant biotech research facilities to focus on the American and Asian markets, and to abandon the development of GM crop varieties for Europe. The EU cultivation authorization request for a Phytophthora-resistant potato variety protected against late blight by resistance genes from wild potatoes, which had successfully passed the R&D phase and shown superior fungal resistance properties in field trials in several EU countries, was withdrawn. This variety could have brought major economical advantages to European farmers and would have reduced the dependency on the fungicides required for potato cultivation, increasing the sustainability of potato production while reducing its environmental footprint [78]. Meanwhile, similar resistant potato varieties are being developed by publicly funded research in Europe, but it is not clear yet how and when these could reach the market.

Due to the perceived prevalent rejection of GM crops by voters, many European politicians have reacted and taken a public stance against products of plant biotechnology. The European authorization system for imports and cultivation of GM crops, which is based on political decisions, has become increasingly unpredictable and dysfunctional [19,80], with an ever-widening gap between the number and timing of authorizations in Europe and the rest of the world that—together with a zero-tolerance policy for traces of unauthorized GM crops—increasingly impedes global commodity imports [12].

Recently, the EU introduced the possibility for member states to ban cultivation of GM crops based on vaguely defined, non-scientific grounds, and the European Commission suggested similar national opt-out possibilities for the import of GM commodities that have been ruled safe by scientific authorities.

#### 3.1.1. National Differences within Europe

Political attitudes towards GM crops are not uniform across all European countries. Rather, EU member states can be separated into three categories depending on their acceptance of plant biotechnology: “adopters”, “conflicted”, and “opposed” member states [12,79]. Among the “adopters” are countries that either grow Bt corn, the only GM crop authorized for cultivation in the EU (Spain, Portugal, Czech Republic, Slovakia, and Romania), as well as member states that would plant GM crops, if varieties suitable to their climatic conditions and farmers’ needs would be available (Denmark, Estonia, Finland, Flanders in Northern Belgium, the Netherlands, Sweden, and the United Kingdom). Governments and industry of “adopter” countries have pragmatic positions and are generally open to GM technologies. As an example, the U.K. government, since 2012, has taken a public position in favor of agricultural biotechnology [12,79].

“Conflicted” member states include countries where scientists, farmers, and the feed industry would support adoption of GM crops, but are resisted by consumers and governments under the influence of political parties and NGOs opposed to genetic engineering. Several countries from this group have cultivated GM crops in the past (France, Germany, Poland), but have since implemented national bans for the planting of insect-resistant Bt Maize line MON810 [2]. As reasons for these politically motivated bans scientific studies of questionable relevance for real-field-situations were cited, marking an increasing trend away from science-based risk-assessment in important European Union member states [81,82]. Political support for plant biotechnology in Germany and France has dwindled over the past few years [12,76]. Additionally, Southern Belgium (Wallonia), Bulgaria, Ireland and Lithuania belong into the “conflicted” group, and follow positions from other countries, especially France and Poland. In Bulgaria, a national ban on the cultivation of Bt maize is in place [2].

Most of the “opposed” member states are located in Central and South Europe (Austria, Croatia, Cyprus, Greece, Hungary, Italy, Malta, and Slovenia); also Latvia can be included into this group. In these countries, most stakeholders and policy makers reject GM crop technology, and only a minority of farmers would consider its use. Public acceptance of GM crops here is lower than the European average [59]. Austria, Greece, Hungary, and Italy have national bans on GM crop cultivation [2]. Organic agriculture and food play an important role in these countries. The skeptical attitude towards GM crop technology is also reflected by regulatory authorities in these countries, e.g., the rather conservative approach of the Hellenic Food Safety Authority [83].

Additionally, Switzerland can be included into the “opposed” group, although it is not a European Union member and was not included in the original classification [12,79] of the member states. In a public initiative in 2005, 55.7% of the participating Swiss voters approved a five-year moratorium on the commercial cultivation of GM crops in Switzerland [84]. An important argument for the moratorium was the perceived requirement for additional scientific data about risks and benefits of GM crop cultivation, to allow a fully informed decision. Within the National Research Program NRP59 “Benefits and risks of the deliberate release of genetically modified plants” (www.nrp59.ch), 30 research projects carried out from 2007 to 2011 analyzed scientific, biosafety, economic and societal aspects of a potential GM crop cultivation in Switzerland. The projects included field trials of GM wheat resistant against mildew, which required large expenditures for security against vandals [85]. The National Research Program did not identify risks specific to the genetic modification for authorized GM crops, and found that some GM crops might offer economic advantages for Swiss agriculture. Co-existence between GM crops and conventional crops in Switzerland would be possible with moderate effort [86]. The Swiss Academies of Arts and Sciences pointed out in a report [87] and an open letter to members of the parliament that some GM plants could improve the sustainability of Swiss agriculture.

These findings, however, had limited effects on the direction of national policy makers. Swiss parliament extended the GM crop moratorium further until 2017 [76], now with the justification of marketing support for GM free products from Swiss agriculture. Even further extensions of the cultivation ban are being discussed. Despite the limited prospects for the growing of GM crops in Switzerland in the foreseeable future, a government-funded protected research site was set up for field trials of GM plants [76,88]. Currently, wheat with improved powdery mildew resistance and cisgenic potatoes with improved late blight resistance have been planted at this research site close to Zurich. It will be interesting to see to what extent these practical examples of GM crops with a sustainability benefit will affect public perception of molecular plant breeding methods.

#### 3.1.2. Europe’s Difficult Environment for Products of Plant Biotechnology

Taken together, the recent trends in many European countries have left Europe with a very difficult environment for GM crops and food. In some countries, such as Germany, public support for plant biotechnology has virtually disappeared, as politicians from all sides of the political spectrum, farmers, the food industry, and retailers have rushed towards the non-GMO camp to take political or commercial advantage, while no one is left or willing to oppose anti GMO-campaigns [89]. The developments over the last decades have led Europe on a path of increasingly negative conditions for GM crops and food which because of path dependency might be difficult to revert or even change [18]. It is difficult to see how consumers’ attitudes towards GM crops and GM food in Europe might change under these conditions, in the absence of possibilities to make own practical experiences with GM food [65], or how a change of attitudes—if it would occur—could have an effect on the cemented negative political and regulatory framework.

### 3.2. USA

For regulations governing market authorization and labeling of food derived from GM crops, the United States has followed a scientific rationale and the concept of “substantial equivalence”. If a novel GM food does not differ in its composition from an established non-GM one, it does not have to be labeled, since this label would give the consumer no relevant information about the physical properties of the product. Only if the GM food has different nutritional properties or allergenicity, the FDA requires labeling [16,18,21,22,25]. In general, the number of US food-related laws is much smaller compared with that of the European Union, and the US legislation concerning GM food can be considered to be more lenient than that of the EU [90]. While a small number of activists protested applications of biotechnology for food production in the U.S., they did not gain wide public support. The majority of consumers were little aware of the increasing use of GM crops for food and feed production in the U.S., and acceptance of the technology was high [17,71]. A first state initiative for labeling of GM food, 2002 in Oregon, was forcefully rejected by over 70% of the voters [91]. The anti-GM crop and food movement appeared to be finished by the year 2008, with GM crops pervasive throughout the U.S. food system and efforts to prevent their use unsuccessful [73].

However, the anti-GMO movement has gained significant momentum in the U.S. since then, and the societal debate about GM crops and food has intensified. The opposition to GM crops and food goes beyond scientific concerns about food safety, and comprises a wide range of environmental and sustainability concerns, as well as socio-economic aspects. It appears that GMOs have become a place for people to focus broader moral concerns about sustainable agriculture, and food in general. A powerful coalition of NGOs, environmental and consumer organizations, proponents of organic agriculture, and “value based” food companies and retailers, demands mandatory labeling for food derived from GM crops, or produced with support from modern biotechnology. The main argument for demanding labels is “a right to know” for consumers, so that they will be able to choose whether to purchase food made with or without GMO ingredients, and thus to increase transparency in the food system. However, the consumer’s right to know can not provide the sole legal basis for mandatory food labeling in the U.S. The majority of the food industry in the U.S. opposes mandatory labeling, since it would unnecessarily increase costs without any benefit for the consumer’s safety, and might in fact reduce consumer choice by pushing GM products off the market, similar to the situation in Europe [21,73,92].

In four states (California in 2012, Washington in 2013, and Colorado and Oregon in 2014) ballot initiatives for the mandatory labeling of GM food were put to public vote, and rejected by a narrow margin. Connecticut and Maine both passed legislation mandating GMO labels, but it is not clear when and whether these will enter into force, since they depend on the introduction of similar regulations in neighboring states. Legislation passed in Vermont (2014) that may make it the first U.S. state to enact the active requirement for GMO labels for food is being contested in court [73]. In the majority of U.S. states, bills have been introduced to require GMO labeling of to prohibit genetically engineered foods.

The consequences of a possible mandatory GM food labeling in the U.S. are difficult to predict. Researchers have pointed at the example of trans-fat labeling, which quickly resulted in the disappearance of products labeled as containing them from the market (although the situations are not directly comparable, since a GMO label would not be based on a proven health concern). Consumer studies showed that a mandatory “contains GMO” label might induce a stronger avoidance of GM products than the presence of voluntarily labeled “does not contain GMO” foods. The real effects of a state-mandated GM label are difficult to assess in such experiments, and it cannot be excluded that they would have a strong signaling effect on consumers, inducing them to avoid GM labeled food [93].

Although a mandatory GMO labeling scheme at federal level in the U.S. seems unlikely and state legislation in this field moves slowly, there has been a strong expansion of voluntary GMO labeling schemes in the U.S. While some retailers are introducing policies to label GMO products in their stores, this is complemented by a broad movement by food producers for non-GMO labeling. The absence of uniform standards for such a labeling and the tendency of some food producers to cash in on a perceived market advantage by using “non-GMO” labels with no or only minor changes to the composition of the product does not contribute much to the transparency for the consumer. It is likely that the importance of voluntary GMO labeling in the U.S. will increase, since it addresses demands from political consumers for more freedom of choice, opens a profitable niche market for retailers, and provides an alternative to mandatory GMO labeling, against which there is strong commercial opposition [72,73]. However, despite of the increasing discussions of agricultural and food applications of biotechnology, but without a mandatory GMO labeling, the USA remain the leading nation for the production of biotech crops, biopharmaceuticals, biomaterials, and bioenergy [80].

### 3.3. China

Already ten years ago, China was seen on the threshold of commercializing GM rice. Results of farm-level pre-production trials with insect-resistant Bt rice had shown higher crop yields and a strongly reduced pesticide use, which also had a positive health impact for farmers [94]. Regulations for mandatory labeling of foods derived from GM crops have been in place since over a decade [21]. The Chinese government invested heavily in biotech crop development, and in 2008 a $3.5 billion GM crop program was announced. In 2009, two Bt rice lines received biosafety certificates for field trials from the Chinese Ministry of Agriculture, which prompted an unusual public outcry by scholars mostly from the humanities and social sciences, and some politicians [45,95]. In the last five years, there has been a shift in attitudes towards GM crops in the population from mostly neutral to negative, and a fierce, increasingly polarized debate about ethical and biosafety issues as well as social issues like ethics, culture, tradition and farmers’ livelihood [96].

Meanwhile, progress towards commercialization of Bt rice appeared to come to a halt. In August 2014, the biosafety certificates fort two Bt rice lines issued in 2009 expired without renewal, which made it illegal to grow these plants and caused speculation whether the Chinese government would cancel further development of GM rice. However, the certificates were renewed for further five years in January 2015 [97]. In February 2015, the government’s No. 1 Central Document, the first major policy document of each year released by the Central Committee of the Communist Party, pledged more government support for research on GM techniques, especially for crops. It lays also the foundation for a new communication campaign, stressing that Chinese scientists must do more to convince a skeptical public of the benefits of GM crops. Researchers are encouraged to enter into a genuine and respectful dialogue with multiple stakeholders and society in general to discuss the true risks and benefits of GM crops [98]. These activities suggest continued support by the Chinese government for biotech crops as an important strategic tool to feed the huge population, but also the recognition that society wants to participate in decisions affecting something as central as food. The authorization for a GM staple food, such as rice in China, would have a strong effect on the global debate about genetically modified food.

### 3.4. India

To supply its large and rapidly growing population with food and income, India needs to increase productivity and efficiency of its agriculture. To promote and support applications of biotechnology, also in agriculture, the Department of Biotechnology (DBT), under the Ministry of Science and Technology, was established in 1986 [99]. India has a thriving agricultural biotechnology research community. The introduction of insect-resistant Bt Cotton for commercial production in 2002 was contested by several groups that claimed a lack of efficiency or even failure of the crop, allegedly driving farmers deeper into debt and even into suicide. However, these general claims did not hold up to scientific scrutiny [100], and Bt cotton cultivation turned out to be a large success. In 2014 about 95% of the cotton area was planted with transgenic varieties [2]. Bt cotton has reduced the dependency on chemical pest control, increased yields and profits for smallholder farmers in a sustainable way over a long period, and has thereby contributed to a positive economic and social development in India. Increased farmer’s income translates also into increased food security for cotton farmers in India [101,102]. Today, over 1000 Bt cotton varieties with different transgenes and genetic backgrounds adapted to local conditions are available. This large selection helps to maintain the agro-biodiversity, the number of different cotton varieties cultivated by farmers has not declined due to the availability of the GM crop [103].

A very different story was the development of insect resistant Bt brinjal (eggplant/aubergine), that carries the same cry1Ac transgene as many Bt cotton hybrids. Brinjal is very sensitive against insect pests, and farmers use large quantities of insecticides and up to 25–80 sprays per season to protect the harvest. In a collaboration between Indian seed company Mahyco, two Universities and two state laboratories, transgenic Bt brinjal was produced in the year 2000. After nine years of extensive agricultural and safety testing including large-scale field trials, the Genetic Engineering Approval Committee (GEAC) of the Ministry of Environment approved hybrids and varieties of Bt brinjal in 2009. However, planting the crop was made illegal by a decision from the Minister of Environment, who rejected the GEAC decision and—after strong pressure from anti-GM activists and farmers, resistance from state governments and consumers and intense discussions in the society—instituted an unlimited moratorium for commercial release. Reasons for this decision were, among others, a widespread mistrust in the state biosafety research and risk assessment, fears of possible health risks of the GM brinjal, and worries about foreign control of the technology and loss of food sovereignty [99,104]. This decision had a strong negative effect on the Indian research community, that had in the course of 15 years developed no less than ten GM plants with different properties, including brinjal, chickpea, sorghum, sugar cane, castor, rice and potato, ready for field trials. However, such trials were no longer authorized, and until 2014 the future for research with GM crops outside confined laboratories was unclear [105,106]. In that year, the new government of Prime Minister Narendra Modi assumed office, and eight Indian states quietly approved field trials for various GM crops. However, more than 20 Indian states and territories still use their power to veto field trials. Progress is limited, the government’s approach to commercial GM crop cultivation (as for the Bt brinjal) seems to be much more cautious than for research, and the need to improve the regulatory framework for biotech crops is obvious [107,108]. While, for the fiber crop Bt cotton agro-economic, considerations seem to be the driving force for the rapid adoption, for the food crop Bt brinjal politics of risk avoidance and public opposition appear to dominate the decision making process [99]. Developments in India are followed around the world, since they reflect discussions and tensions concerning the use of GM crops in many developing countries that have to balance the need for increased agricultural productivity with opposition to new technologies within the society [108].

## 4. Outlook: New Breeding Techniques and Consumer Attitudes

Plant biotechnology is a rapidly evolving field, and developments have sped up substantially with the availability of molecular tools as basis for new breeding techniques. Intragenesis and cisgenesis, where only genetic information from the same species but no “foreign genes” are transferred [109]; and new precise genome editing tools such as the CRISPR/Cas9 endonuclease system [110,111,112] have expanded the possibilities for crop breeding. They contribute to the large number of biotech plants with novel or further improved traits in the R & D pipeline [113,114].

Given the public skepticism about crops modified by traditional genetic engineering approaches, such as transgenesis, and the high regulatory hurdles for GM crops in agriculture, many researchers and seed companies hope that the new breeding techniques will facilitate consumer acceptance and the way to market for plants with improved traits, thereby circumventing the roadblocks transgenic GMOs have faced in many countries [110,115]. Indeed, plants developed with the help of the new breeding techniques do not fit the traditional definition of GMOs, and in many places, political discussions are ongoing on how they should be regulated. This regulatory uncertainty, while opening an opportunity for the development of a more science-based approach focusing less on the breeding process but on the properties of a new plant variety, also is an impediment to the further application of these techniques and stifles innovation [111,112,116].

Will consumers view plants developed by the new breeding techniques as more acceptable? One point of contention with traditional transgenic GM crops is the presence of “foreign” genetic information, derived from other species, which is sometimes perceived to be against the natural order, or is suspected to cause unwelcome effects by horizontal gene transfer, or by ingestion [52,117]. Plants produced with the new breeding techniques do not contain genes transferred across species boundaries, and the genomic changes by modern genome-editing tools often are indistinguishable from those present in plants developed by classical breeding. Nevertheless, a technical procedure, which might be perceived as unnatural, is involved in producing these new plants.

When European consumers were asked in the Eurobarometer survey in 2010 about their attitude towards the transfer of disease resistance genes from wild apples to cultivated varieties, without “foreign” DNA, encouragement for these cisgenic apples was clearly higher (46%) than for transgenic apples (29%), but still the majority of respondents (52%) considered them unnatural, and 40% felt uneasy about them. Only a minority of respondents differentiate their support for transgenic and cisgenic apples, and more than three quarters demand that even cisgenic apples be labeled as GM food [33,35,117]. Although respondents from five European countries were prepared to pay more for rice labeled “cisgenic” than if it was labeled as “genetically modified”, only 36% or 38%, respectively, were willing consume rice improved by biotechnology [118]. In addition, NGOs opposing genetic engineering, including Greenpeace and Friends of the Earth Europe, are warning that some of the new breeding techniques allow more radical changes to plant genomes than currently used genetic engineering methods, that risk assessment for these new technologies is not yet possible, and that therefore the precautionary principle should be applied. Additionally, they demand that all food products derived using new breeding techniques should be labeled as “genetically modified” [119]. Given the large historical influence of NGOs—at least in Europe—on shaping consumers’ attitudes and the biotech regulatory landscape [16,24,25], it is not clear yet whether the future regulatory framework for new breeding techniques will be conductive to their further development, and whether products derived from them will be embraced by consumers. Whereas, in the U.S., authorities so far have considered plants derived from cisgenesis and genome editing not as GMOs, the jury is still out in Europe. Recently, a controversy has erupted in the EU concerning a herbicide-tolerant oilseed rape by the U.S. company Cibus, created by oligonucleotide-directed mutagenesis of a few genomic nucleotides. While these plants will be grown in the United States already in 2015 and a cultivation authorization from Canada also has been obtained, their legal status in Europe is unclear. After the German Federal Office of Consumer Protection and Food Safety (BVL) had declared in spring 2015 that, based on expert’s opinion, it would not consider the Cibus oilseed tape as a GMO, massive protests from NGOs tried to reverse that decision [120]. Meanwhile, the European Commission has asked member states to refrain from regulatory decisions concerning new breeding techniques while a political position for the EU on the definition of GMOs is being developed. According to the Commission, this analysis is currently ongoing and results cannot be anticipated yet.

It is becoming increasingly clear that the present process-based regulatory framework for GM crops, which has been developed over a decade ago when these new breeding techniques were not available yet and that focuses on the technology used to produce a new plant variety and not on the product properties, is no longer fit for the purpose [79]. The introduction of stringent authorization requirements here would hinder innovation in the plant-breeding field, and would undermine consumers’ confidence in the new technologies [12,111]. Hence, a too rigid regulatory framework for new breeding techniques might prevent developments whose results might be welcomed by consumers. New breeding techniques may facilitate the development of plant varieties with increased disease and pathogen resistance which are less dependent on plant protection products and hence would contribute to the development of more sustainable and “natural” agricultural systems, something consumers clearly desire [121].

## 5. Conclusions

When discussing customer acceptance of GM crops, different cases have to be distinguished. Farmers themselves are customers of seed companies and distributors. Due to clear advantages to them, their adoption and hence acceptance of GM crops in many world regions where biotech seeds are available is very high. Additionally, in international commodity trade, the acceptance of GM plant varieties—mostly used as feed—is broad, and certified non-GMO commodities represent only a niche market. On the other hand, consumers in many countries have doubts about possible risks and benefits of GM crops and food, and stated acceptance among this customer class for GM food products is lower, especially in Europe. Consumers’ attitudes take many factors into account, including information, trust, beliefs, perceptions of risks and benefits, and develop on the background of a complex set of personal values that to a large degree predetermine how external information is processed and evaluated.

Therefore, a rejection of genetically engineered plants and products derived from them often is not based just on clearly defined arguments. Rather, GM crops have become a lightning-rod for negative emotions caused by everything that is considered bad about modern agriculture and the food system, worries about the place of the individual in modern society, feelings of limited individual self-determination *versus* the increasing influence of large corporations, and concerns about misguided economic developments, globalization, and growing inequality. This makes many discussions about GM crops difficult or even futile, since apparently they revolve around disputed “scientific” facts, like possible effects of a GM plant on the environment or whether a GM plant might have unexpected health impacts, but in reality they are about fundamental differences in the underlying values of the discussion participants, which may be impossible to reconcile.

That consumers’ attitudes and behavior are not the result of purely logical processes is illustrated, for example, by the discrepancy between the facts that the large majority of the many millions of tons of soybeans imported annually into the EU as feed are genetically modified varieties, on the one hand, and the stated strong rejection of GM crops by many consumers on the other. Information about the ubiquitous use of GM crops as feed for animal food production in Europe is readily available to anyone who wants to know, but most consumers choose to ignore this issue. Only a minority actively tries to avoid food produced with the help of GM crops, by buying organic food or from labels that renounce GM feed. Thus, the apparently deep conviction with which many consumers in Europe reject GM crops has remarkably little consequences on their actual behavior.

What are the chances for changes to the deeply engrained negative attitudes of consumers towards GM crops and food in Europe? The current situation in many countries is not encouraging. Politicians of all colors, food companies and retailers have taken opportunistic positions critical of GMOs, public support for them has largely disappeared, and few have the courage to speak out in favor of them. Almost no GM food products are found on European grocery shelves, so consumers have no chance to make practical experiences with them—which would be an important contribution to an attitude change. Environmental NGOs have realized this, and make sure that the situation stays unchanged, by putting strong pressure on retailers to exclude authorized, safe and correctly GM labeled products from their product line. An increasingly dysfunctional, politicized authorization system and onerous regulatory requirements make bringing a new GM crop or food product to the market less and less attractive. The withdrawal of plant biotech companies’ R & D from Europe due to unfavorable framework conditions diminished the innovation potential of European commercial plant research, and reduced the chances that GM crops tailored to European farmers’ needs will be developed. Academic research for the development of GM crops meets with resistance in many places, too. So, also the negative development for plant biotech research in Europe contributes to the absence of GM crop or food products that could convince consumers to change their minds. A change in this increasingly hardened political and societal situation will unlikely come from the consumers’ side, but rather derive from economic or ecologic pressures for which GM crops might provide a solution, and it will require considerable political courage.

In the U.S., the long dormant discussion about GM crops and foods has increased in intensity. Political initiatives for mandatory GM labeling and voluntary GM-free labeling schemes have brought this topic to the attention of many consumers, and it is possible that the niche market for food produced without support from genetic engineering will grow. However, it is unlikely that in a country where many foods since many years have ingredients derived from GM crops the wheel can be turned back completely and U.S. consumers’ attitudes will become as hostile towards GM food as those of the Europeans, where labeled GM foods have been kept off the market.

Interesting will be the developments in China, which tries to balance the need for modern technologies including GM crops to support a more productive and sustainable agriculture with an increasing societal discussion about their benefits and drawbacks. Here, decision-makers have shown a growing openness and willingness to listen to consumers’ concerns, and have realized that a decision to authorize GM foods without support in the society would be difficult.

Consumer attitudes globally will have an increasing impact on the development of plants with new and improved properties, either by classical genetic engineering or by the new breeding techniques, including cisgenesis and genome editing. These developments will also affect the future of crop plants genetically modified to resist virus infection, many of which are at the research and development stage. Bean golden mosaic virus (BGMV) resistant RNAi-beans recently were approved for commercial use by the Brazilian technical biosafety commission CTNBio, and papaya ringspot virus (PRSV) resistant transgenic papaya have been on the market since 1998 [4,122,123]. Consumers’ decisions will determine which products will be successful in the market, and their political decisions will shape the regulatory framework for the development and application of new technologies. How can an apparently increasing skepticism of consumers and society in general towards modern plant breeding technologies be reconciled with the need to develop plants that contribute to a more productive and at the same time more sustainable agriculture, using the full toolbox available to plant breeders? One important objective would be to move beyond the GM debate [74]. Only by leaving the old patterns of the “pro/contra-GM” discussion, by focusing less on by which technologies a new plant property was introduced but more on the advantages and possible drawbacks of the new properties themselves and on the common aims will it be possible to address the challenges of global food security and agricultural sustainability in a productive manner. This discussion should also acknowledge and openly debate fundamental differences in the values that contribute to the development of individual attitudes. This does not necessarily resolve the conflict, but makes clear what is at the core of the dispute, thus clarifying the opposing views and possible criteria for decisions [124]. Finally, the political framework should support the right of consumers to make individual decisions—even if these are not based solely on “scientific facts”, but take into account more broad discussions about which forms of life we want to live and which innovations are considered desirable or problematic by society. The inclusion of these democratic elements into the discourse should help making truths more robust and contribute to the sharing of responsibility for future developments between all elements of society, including science as one integral part of it [125]. Hopefully, these discussions will eventually contribute to a societal framework that will not block, but enable innovations that might benefit society as a whole.
